# Use of National Database of Health Insurance Claims and Specific Health Checkups for examining practical utilization and safety signal of a drug to support regulatory assessment on postmarketing drug safety in Japan

**DOI:** 10.3389/fmed.2023.1096992

**Published:** 2023-02-23

**Authors:** Haruka Shida, Kazuhiro Kajiyama, Sono Sawada, Chieko Ishiguro, Mikiko Kubo, Ryota Kimura, Mai Hirano, Noriyuki Komiyama, Toyotaka Iguchi, Yukio Oniyama, Yoshiaki Uyama

**Affiliations:** ^1^Office of Medical Informatics and Epidemiology, Pharmaceuticals and Medical Devices Agency, Tokyo, Japan; ^2^Office of Pharmacovigilance I, Pharmaceuticals and Medical Devices Agency, Tokyo, Japan; ^3^Office of Pharmacovigilance II, Pharmaceuticals and Medical Devices Agency, Tokyo, Japan

**Keywords:** real-world data, pharmacoepidemiology, national claims database, drug safety, regulatory decision-making

## Abstract

The Pharmaceuticals and Medical Devices Agency (PMDA) has conducted many pharmacoepidemiological studies for postmarketing drug safety assessments based on real-world data from medical information databases. One of these databases is the National Database of Health Insurance Claims and Specific Health Checkups of Japan (NDB), containing health insurance claims of almost all Japanese individuals (over 100 million) since April 2009. This article describes the PMDA’s regulatory experiences in utilizing the NDB for postmarketing drug safety assessment, especially focusing on the recent cases of use of the NDB to examine the practical utilization and safety signal of a drug. The studies helped support regulatory decision-making for postmarketing drug safety, such as considering a revision of prescribing information of a drug, confirming the appropriateness of safety measures, and checking safety signals in real-world situations. Different characteristics between the NDB and the MID-NET^®^ (another database in Japan) were also discussed for appropriate selection of data source for drug safety assessment. Accumulated experiences of pharmacoepidemiological studies based on real-world data for postmarketing drug safety assessment will contribute to evolving regulatory decision-making based on real-world data in Japan.

## 1. Introduction

Spontaneous reports of adverse drug reactions are a valuable source for postmarketing drug safety assessments in Japan (1). However, it is also known to have some limitations in drug safety assessment based on these reports such as underreporting, more serious cases frequently reported and a lack of denominator (2, 3). Meanwhile, in recent years, secondary utilization of data from the database has received significant attention for the regulatory purpose of postmarketing drug safety assessment in accordance with advancements in the medical information database infrastructure and further advancement of regulatory assessment of drug safety in Japan (4). The Pharmaceuticals and Medical Devices Agency (PMDA) has conducted pharmacoepidemiological studies for postmarketing drug safety assessment based on real-world data (RWD) from medical information databases (5). One of those databases is the National Database of Health Insurance Claims and Specific Health Checkups of Japan (NDB), which was established by the Japanese Ministry of Health, Labour, and Welfare (MHLW) based on “Act on Assurance of Medical Care for Elderly People” (6). The NDB is one of the world’s largest health-related databases and contains nationwide electronic health insurance claims data and specific health check-up data gathered from all medical institutions, including hospitals, clinics, pharmacies, and dental clinics, and covers the medical information of almost all Japanese individuals (over 100 million) since April 2009 (6).

This article describes PMDA’s regulatory experiences in utilizing the NDB for postmarketing drug safety assessment, especially focusing on the recent cases of use of the NDB for examining the practical utilization and safety signal of a drug. The permission to use the NDB for drug safety assessment has been granted to the PMDA by the expert committee of MHLW since March 2016.

## 2. Study 1: Drug utilization study of triptan-based drugs in assessing a drug-overuse headache

### 2.1. Background and objective

Drug-overuse headache was a concern for triptan-based drugs, a class of migraine medication, based on Japanese and foreign spontaneous reports. In recent years, although the prevalence of triptan overuse has been elucidated gradually based on healthcare databases in foreign countries (7), no data were available on the real-world situation of drug use in clinical practice in Japan. This study aimed to identify the practical use and prescription patterns of triptan-based drugs, including their overuse.

### 2.2. Methods

We utilized the NDB to identify patients who were prescribed triptan-based drugs, including eletriptan hydrobromide, sumatriptan succinate, sumatriptan, zolmitriptan, naratriptan hydrochloride, and rizatriptan benzoate, between August 1, 2010, and March 31, 2016. For each patient, the total monthly dose equivalent of sumatriptan succinate was calculated based on the single or maximum daily dose of each triptan-based drug. The definitions to identify a patients with triptan-overuse were created based on the diagnostic criteria of “Triptan-overuse headache” in the International Classification of Headache Disorders 3rd edition (ICHD-3) (i.e., regular intake of one or more triptans, in any formulation, on ≥10 days/month for >3 months) (8). Actually, in order to consider the amount of intake in the prescription data, the definition was slightly modified with two variations. The first definition intended for “suspected overuse” and was defined as a patient who met at least one of the following criteria; (1) the total monthly dose exceeded 10 times the single dose of sumatriptan succinate for four or more consecutive months, or (2) the total monthly dose in a certain month exceeded four or higher integral multiple (*X* ≥ 4) of 10 times the single dose of sumatriptan succinate and additionally prescribed triptan-based drugs within X months after. The second definition intended for “overuse” and was defined by changing the term “10 times the single dose of sumatriptan succinate” in “suspected overuse” to “10 times the maximum daily dose of sumatriptan succinate.” It should be noted that these definitions were used for this study without validation because of the exploratory nature of the study purpose. All analyses were performed by using SAS 9.4 statistical software (SAS Institute, Cary, NC, USA).

### 2.3. Results and discussion

Overall, 2,078,556 patients with triptan-based drug prescriptions were included in the analysis. Of those, 102,871 (4.95%) were “suspected overuse” and 16,426 (0.79%) were “overuse” cases. These results indicate that there were a certain number of patients with suspected overuse of triptan-based drugs in Japan. MHLW/PMDA evaluated the results of this study as well as the situations in foreign countries in consultation with external experts and revised the important precautions section and the clinically significant adverse reactions section in the package inserts (PIs) of triptan-based drugs to include information about a medication-overuse headache (9).

## 3. Study 2: Drug utilization study of valsartan-containing drugs in the assessment of carcinogenic contaminant

### 3.1. Background and objective

In July 2018, valsartan-containing drugs approved for hypertension were voluntarily recalled by the marketing authorization holder (MAH) due to contamination with an impurity, N-nitrosodimethylamine (NDMA), a known carcinogen (10). More recently, no potential relationship between cancer risk and carcinogenic contaminant during valsartan prescription have been reported (11), but there was little safety information available at the time of this study in Japan. This study aimed to elucidate the prescribing patterns and cumulative dose per patient of valsartan-containing drugs to provide information related to the assessment of patient health effects caused by NDMA.

### 3.2. Methods

We utilized the NDB to identify patients with prescriptions for recalled products between June 1, 2014 and March 31, 2016. We estimated the cumulative dose per patient based on each prescription’s daily dose (mg) and period (days). We then calculated summary statistics, including mean, standard deviation, median, interquartile range (IQR), and minimum and maximum values for the cumulative dose, and illustrated the frequency distribution chart. All analyses were performed by using SAS 9.4 statistical software (SAS Institute, Cary, NC, USA).

### 3.3. Results and discussion

**FIGURE 1:**
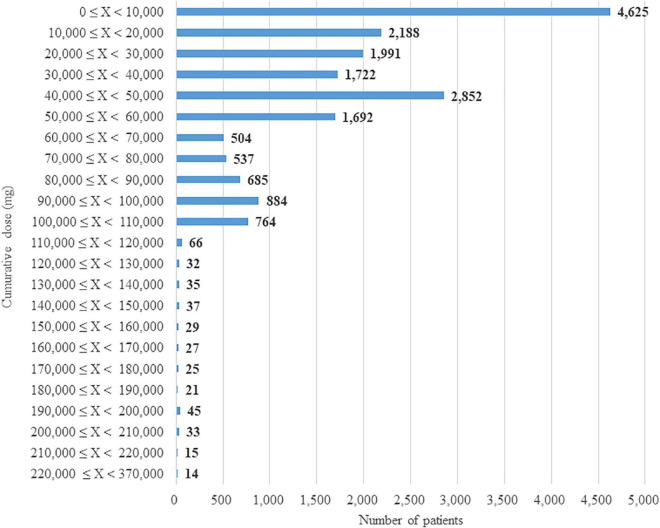
Frequency distribution for cumulative dose of valsartan-containing drugs.

A total of 18,823 patients with prescriptions for recalled products were identified. The median cumulative dose was 33,600 mg (IQR: 10,080–51,920 mg), with minimum and maximum values of 40 mg and 360,000 mg, respectively. As shown in the frequency distribution chart of Figure 1, if the maximum daily dose (160 mg in Japan) of valsartan-containing drugs was prescribed for the entire study period (670 days), the maximum cumulative dose was assumed to reach at the range of 100.000-110,000 mg. The 764 patients fell into this maximum range, and 98% out of the total patients were under this range. The study elucidated the prescribing patterns and cumulative dose per patient of the recalled products and provided useful information related to assessing patient health effects caused by NDMA.

## 4. Study 3: Drug utilization study of metformin-containing drugs in the assessment of carcinogenic contaminant

### 4.1. Background and objective

In December 2019, the Health Sciences Authority (HSA) in Singapore announced that certain metformin-containing drugs approved for diabetes were voluntarily recalled by MAH owing to contamination with NDMA impurities (12). Similar contamination with NDMA was found in metformin-containing drugs marketed in Japan (13, 14). Based on the National Institute of Health Sciences (NIHS) risk assessment in Japan, the MHLW concluded that no additional safety-related regulatory actions were required (15). This study aimed to examine the daily dose and cumulative prescription days of metformin-containing drugs in a real-world setting to support the assumptions used in the risk assessment.

### 4.2. Methods

We utilized the NDB to identify patients who were prescribed metformin-containing drugs between August 1, 2010 and March 31, 2018, and examined the earliest date of prescription of metformin-containing drugs per patient as the first prescription. We then excluded patients with first prescriptions between August 1, 2010, and March 31, 2011. As the date of the first prescription affects both the follow-up period and cumulative prescription days, we divided the target population into seven sub-cohorts based on the fiscal year of the first prescription date. We calculated the cumulative number of prescription days per patient and its summary statistics for each sub-cohort. In the case of overlapping prescription periods for consecutive prescriptions, days of overlap were ignored. Moreover, we calculated the daily dose per patient by dividing the cumulative dose by cumulative prescription days and its summary statistics for each sub-cohort. All analyses were performed by using SAS 9.4 statistical software (SAS Institute, Cary, NC, USA).

### 4.3. Results and discussion

A total of 4,239,798 patients with prescriptions of metformin-containing drugs were identified in the main cohort. The median daily dose was 708.4 mg (IQR: 509.1–916.0 mg), and the daily doses for approximately 97% of patients were <1,500 mg. Table 1 shows the number of patients and the summary statistics of the cumulative prescription days for each sub-cohort. The third quartile of cumulative prescription days was approximately 6 years in patients in the sub-cohort for 2011 which was the longest follow-up period of up to 7 years. These results indicate that in most patients, metformin-containing drugs have been continually prescribed for nearly a decade in Japan. The results of this study supported the appropriateness of the risk assessment by NIHS, showing that the daily dose of metformin-containing drugs in clinical practice was consistent with the assumption of the risk assessment (1,500 mg/day) (15).

**TABLE 1:** Number of patients and summary statistics of cumulative prescription days of metformin-containing drugs for each sub-cohort.

Fiscal year	*N*	Median (days)	IQR (days)
2011	439,441	1,512	459–2,161
2012	366,377	1,402	445–1,845
2013	399,427	1,248	438–1,528
2014	377,824	1,019	404–1,206
2015	392,860	735	341–874
2016	397,498	435	269–562
2017	426,947	139	60–244

IQR, interquartile range.

## 5. Study 4: Detection of safety signal for retinal detachment by fluoroquinolones

### 5.1. Background and objective

Inconsistent findings regarding the increased risk of retinal detachment (RD) associated with fluoroquinolones (antibiotics), have been reported (16–20). Under these circumstances, the Pharmacovigilance Risk Assessment Committee of the European Medicines Agency recommended in 2014 to update the product information of fluoroquinolones to warn prescribers regarding impaired vision in the section of special warnings and precautions for use in consideration of the severity of RD (21). In Japan, there have been no spontaneous reports regarding the relationship between RD and fluoroquinolones as of July 2019, and recent report based on the healthcare database shows no association between fluoroquinolones and RD (22). This study aimed to check the safety signal indicating the association between RD and fluoroquinolones with reference to those of other antibiotics in Japan.

### 5.2. Methods

In utilizing the NDB, a sequence symmetry analysis (SSA) (23) was conducted in patients with both prescriptions of antibiotics (fluoroquinolones, cephems, macrolides, penicillins, penems, aminoglycosides, or tetracyclines) and the surgery for RD between April 1, 2011, and March 31, 2016. Surgery for RD was defined as the combination of diagnosis and surgical procedure in the same month. Patients whose dates of the first exposure and first outcome were within 90-days were included. Patients who met at least one of the following criteria were excluded: (1) exposure or outcome that occurred from August 1, 2010 to March 31, 2011; and (2) the first exposure and the first outcome occurred on the same date. The crude sequence ratios (CSRs), the number of patients prescribed with an antibiotic prior to RD divided by the number of patients diagnosed as RD before prescribing antibiotics and the adjusted sequence ratios [ASRs, CSR divided by null-effect sequence ratio (23)] were calculated with their 95% confidence interval (CI). These parameters were calculated based on patient numbers accounted for each administration route of fluoroquinolones prescription (i.e., “systemic fluoroquinolones,” “topical fluoroquinolones except for ophthalmic use,” and “ophthalmic fluoroquinolones”). Note that “ophthalmic fluoroquinolones” was independently categorized because some dosage forms of fluoroquinolones were approved and used for the perioperative sterilization of the ocular, which may be a confounding factor for RD. All analyses were performed by using SAS 9.4 statistical software (SAS Institute, Cary, NC, USA).

### 5.3. Results and discussion

A total of 81,686 patients with prescriptions of fluoroquinolones were identified, and the ASR for fluoroquinolones and RD was 2.76 (95% CI: 2.72–2.80); none of the other antibiotics increased the ASR (Supplementary Table 1). As shown in Table 2, the increased ASR was observed only for ophthalmic fluoroquinolone but not for other route administrations, such as systemic and other topical (i.e., percutaneous) administrations. The identified signal in the ophthalmic fluoroquinolone was also not likely associated with fluoroquinolone because ophthalmic fluoroquinolones are usually prescribed for perioperative sterilization before surgery for RD. PMDA evaluated these results as well as other relevant information, such as spontaneous adverse event reports in Japan, and concluded that no additional safety measures were required at that time (in January 2020).

**TABLE 2:** Crude sequence ratio and adjusted sequence ratio of use-specific fluoroquinolones.

	Total number of patients	CSR	ASR (95% CI)
Systemic fluoroquinolones	22,253	0.99	0.97 (0.95–1.00)
Topical fluoroquinolones except for ophthalmic use	3,860	1.07	1.06 (0.99–1.13)
Ophthalmic fluoroquinolones*	105,265	2.98	2.91 (2.87–2.95)

Dates of the first exposure and first outcome were within 90-days. *Ophthalmic fluoroquinolones which were used for ophthalmologic pre-operative period. ASR, adjusted sequence ratio; CI, confidence interval; CSR, crude sequence ratio.

## 6. Discussions

The results of four pharmacoepidemiological studies using the NDB helped support regulatory decision-making for postmarketing drug safety, such as considering a revision of prescribing information, confirming the appropriateness of safety measures, and checking safety signals in real-world situations. These studies have further enriched our knowledge and experiences in utilizing NDB for regulatory purposes and have expanded the possibility of NDB utilization in drug safety assessment, in addition to studies for a particular safety risk assessment of a drug compared with other drugs (24–26).

**FIGURE 2:**
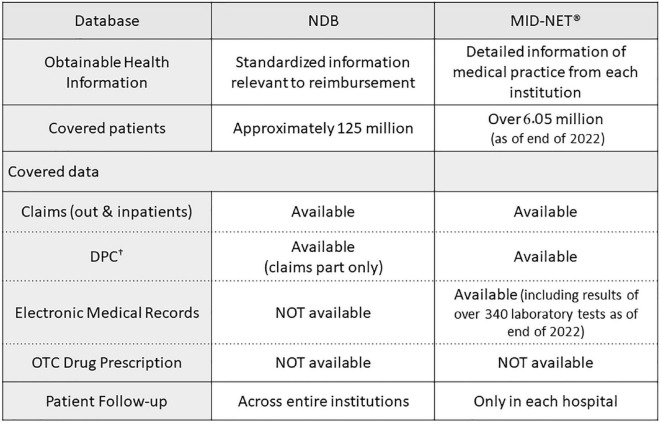
Comparisons of characteristics between NDB and MID-NET^®^. ^†^Known as the diagnostic procedure combination (DPC) data, similar to diagnosis related groups (DRGs) in USA. (Acute Inpatient Medical Care System data).

In addition to studies using the NDB described in this manuscript, PMDA has also conducted various studies using another database called the Medical Information Database Network (MID-NET^®^), which is known as a reliable and valuable database in Japan (27, 28), for postmarketing drug safety assessment. As shown in Figure 2, NDB and MID-NET^®^ have different characteristics in terms of data categories and patient follow-up. For example, the NDB is the largest database with nation-based medical claims and allows the follow-up of a patient regardless of their visiting hospitals and types of health insurance (6). In contrast, the MID-NET^®^ includes not only claims data but also electronic medical records with laboratory test results in collaborative hospitals, although a patient can only be followed within a hospital (27, 28). For all four studies presented in this article, it was necessary to identify patients to the extent possible for understanding the real-world situation of drug use. For the study for detection of safety signal, the outcome of RD was defined only with claims data but not with laboratory test results. Therefore, we selected the NDB as a data source for these studies. MID-NET^®^ is usually selected for a study in which laboratory test results are necessary for analysis (29–32). As described above, an understanding of the characteristics of each database is important to select a data source fit to a study purpose. Accumulating practical experiences in utilizing these databases in PMDA will enable more accurate database studies and increase the confidence of obtained real-world evidence (RWE) to be used as a basis for regulatory decision-making.

In conclusion, the PMDA will continue to utilize the NDB for conducting pharmacoepidemiological studies based on RWD for postmarketing drug safety assessment. Accumulated experiences through those studies will contribute to evolving regulatory decision-making based on RWE.

## Data availability statement

The original contributions presented in this study are included in the article/Supplementary material, further inquiries can be directed to the corresponding author.

## Ethics statement

Since these studies were conducted as an official activity of the PMDA under the Pharmaceuticals and Medical Devices Agency Law [Article 15–5–(c) and (f)] (33), they were not subject to review by the institutional review boards (25). Written informed consent from the participants’ legal guardian/next of kin was not required to participate in this study in accordance with the national legislation and the institutional requirements.

## Author contributions

HS, SS, CI, MK, RK, MH, NK, and YU designed the studies. HS, SS, and KK performed the analysis and analyzed the data. HS, SS, KK, CI, MK, RK, MH, NK, TI, YO, and YU wrote the manuscript. All authors contributed to the final manuscript, read and approved the submitted version.
