# Supplementary material for: Use of National Database of Health Insurance Claims and Specific Health Checkups for examining practical utilization and safety signal of a drug to support regulatory assessment on postmarketing drug safety in Japan
Source: Front Med (Lausanne). 2023 Feb 23;10:1096992. doi: 10.3389/fmed.2023.1096992 (PMC9995365; doi:10.3389/fmed.2023.1096992)
Supplement: Supplementary file 1 [file Table_1.pdf]

Table S1. Crude sequence ratio and adjusted sequence ratio of antibiotics

	Total number of patients	CSR	ASR (95%CI)
Fluoroquinolones	81,686	2.86	2.76 (2.72－2.80)
Cephems	51,006	0.96	0.93 (0.91－0.95)
Macrolides	16,747	1.12	1.09 (1.06－1.13)
Penicillins	10,358	0.93	0.93 (0.89－0.96)
Penems	3,866	0.98	0.97 (0.91－1.04)
Aminoglycosides	15,287	0.69	0.67 (0.65－0.69)
Tetracyclines	2,950	1.02	1.01 (0.94－1.09)

Dates of the first exposure and first outcome were within 90-days

ASR: adjusted sequence ratio, CI: confidence interval, CSR: crude sequence ratio
